# Coping With Oral Tongue Cancer and COVID-19 Infection

**DOI:** 10.3389/fpsyt.2021.562502

**Published:** 2021-06-16

**Authors:** Rita De Berardinis, Paolo Guiddi, Sara Ugolini, Francesco Chu, Giacomo Pietrobon, Gabriella Pravettoni, Fabrizio Mastrilli, Susanna Chiocca, Mohssen Ansarin, Marta Tagliabue

**Affiliations:** ^1^Division of Otolaryngology and Head and Neck Surgery, IEO, European Institute of Oncology, Istituto di Ricovero e Cura a Carattere Scientifico, Milan, Italy; ^2^Applied Research Unit for Cognitive and Psychological Science, European Institute of Oncology, Istituto di Ricovero e Cura a Carattere Scientifico, Milan, Italy; ^3^Department of Otorhinolaryngology, Istituto di Ricovero e Cura a Carattere Scientifico Fondazione Policlinico San Matteo, University of Pavia, Pavia, Italy; ^4^Department of Oncology and Hemato-Oncology, University of Milan, Milan, Italy; ^5^Medical Administration, Chief Medical Officer, IEO, European Institute of Oncology, Istituto di Ricovero e Cura a Carattere Scientifico, Milan, Italy; ^6^Department of Experimental Oncology, IEO, European Institute of Oncology Istituto di Ricovero e Cura a Carattere Scientifico, Milan, Italy; ^7^Department of Biomedical Sciences, University of Sassari, Sassari, Italy

**Keywords:** oral tongue cancer, psychological distress, Coronavirus SARS-CoV-2, loneliness, coping, telemedicine

## Abstract

To date, April 19, 2021, the coronavirus disease 2019 (COVID-19) caused about 140,886,773 confirmed cases and more than 3,000,000 deaths worldwide since the beginning of the pandemic. Oncology patients are usually frail due to the fear of prognosis, recurrence, and outcomes of treatments. Thus, coping with cancer is a complicated process that is necessary to overcome oncological challenge, even more in case of Severe Acute Respiratory Syndrome Coronavirus 2 (SARS-CoV-2) disease. This is a brief case report on a middle-aged man affected by advanced oral tongue cancer and COVID-19, describing his experience of cancer diagnosis, surgical treatment, and rehabilitation during the hospital quarantine for COVID-19. Besides the traumatic experience due to the functional alteration in breathing, eating, and speaking caused by major surgery and the concurrent facial disfigurement, our patient had to face a COVID-19 diagnosis, which implied hospital and social isolation. The aim of this perspective work is to focus on the role of the psychological support in the management of hospital distress related to COVID-19 psychophysical loneliness or alienation. In our experience, such support should anticipate patients' oncological surgery or treatment and should be implemented through telemedicine in case of isolation or after hospital discharge.

## Introduction

To date, April 19, 2021, the coronavirus disease 2019 (COVID-19) has caused 140,886,773 confirmed cases and 3,000,000 deaths worldwide, since the beginning of the pandemic (1). In the current international health emergency caused by Severe Acute Respiratory Syndrome Coronavirus 2 (SARS-CoV-2), the whole healthcare system has been facing an unprecedented crisis: both medical and psychological aspects are continuously evolving.

During the current pandemic, in some hospitals oncological treatments have continued despite the lack of places in intensive care units and surgical theaters due to the high number of severe COVID-19 cases (2–8).

Oral tongue carcinoma is a rare tumor but the most common cancer of the oral cavity, and surgery remains the gold standard of treatment (9). Among head and neck cancers, tongue neoplasm is surely the most psychologically traumatic cancer (10) due to the distressing alteration of essential functions. These include eating, swallowing, breathing, speech, loss of taste and smell, impaired sensitivity, xerostomia, residual pain, swelling, and facial disfigurement (11, 12). Moreover, disease and treatment cause heavy disabilities and patients often have to face extensive rehabilitative programs, consisting of speech therapy, swallowing, and dental/maxillofacial restoration, as well as physical and occupational therapies (13).

The diagnosis of oral cancer is one of the most psychologically traumatic events to experience (14). Among other disturbing (or afflicting) aspects, social isolation, and altered perception of the future perspectives are of utmost importance.

Although recently, most attention has been on the current pandemic, the incidence of all other diseases has not decreased, and COVID-19 has in part exacerbated medical and psychological conditions which were already in a precarious balance.

## Article Types

This study is a perspective, case report presentation. The study was reviewed by the European Institute of Oncology (IEO) Ethic Committee (IEO code 2432), and an informed consent for confidentiality, privacy, and data protection was obtained by the patient.

### Medical Case Presentation

On March 9, 2020, the day after Italy was placed in a national strict “lockdown” due to exponential increase of COVID-19 cases, a 50-year-old male patient was admitted to the Division of Otolaryngology and Head and Neck Surgery Division at the European Institute of Oncology in Milan for advanced oral tongue cancer treatment (15). He had no history of smoking and alcohol habits and no psychiatric records. According to the international guidelines for his disease stage, he underwent major surgery: right glossectomy type IIIA plus en-block ipsilateral neck dissection, temporary tracheotomy, and reconstruction of the oral defect with a radial forearm free flap. Surgery lasted for 12 h (16). On the second postoperative day, a naso-oropharyngeal swab and a bedside chest X-ray were performed for persistent fever and episodes of mild hypoxemia, revealing SARS-CoV-2 pulmonary infection. Thus, the patient was placed in droplet isolation and the hospitalization time was doubled. During his quarantine, the patient also requested psycho-oncological support. The patient's social isolation continued for 6 weeks after hospital discharge, due to persistent viral positivity of nasopharyngeal swabs, further worsening his psychological frailty.

### Psychological Consequences of COVID-19 After Head and Neck Major Surgery

The usual postoperative course for patients undergoing major surgery for tongue cancer is very complicated: speaking is not allowed, head mobilization is generally impaired, and face edema and scars represent difficult steps to overcome. Oral feeding is usually avoided until the 7th postoperative day, if no complication occurs, and swallowing and speech rehabilitation are very challenging.

For these reasons, even during the “non-pandemic era” patients affected by oral cancer could easily enter into a depressive status. In oral cancer patients, depressive symptoms are closely associated with treatment adherence, self-care behaviors, re-socialization, and aspects of resilience (13). Depression and fear of cancer recurrence and future life conditions are psychological aspects which commonly characterize the postoperative phase (14, 17, 18).

Consequently, patients have to face their conditions through coping strategies. Coping is “a cognitive and behavioral effort to manage specific external and/or internal demands that are appraised as taxing or exceeding the resources of the person” (19, 20).

Clearly, COVID-19 can only worsen the physical and psychological challenges which usually arise in an oral cancer patient.

During the COVID-19 pandemic, our Hospital applied several measures (8). Above all, patients' family members and friends were not allowed to visit because of the risk of virus infection during postsurgery rehabilitation (8). Unfortunately, several studies confirm that isolation affects patients' adherence to self-care and rehabilitation schedules, decreasing their motivation (21–25). This clinical and social scenario exposes patients to a higher risk of psychological burden, as recently published (23, 26).

## Discussion

All discussed health-related conditions affected the normal coping strategies of our patient and increased his vulnerability (27).

During the postoperative rehabilitation, the patient was troubled by the fear of his oncologic disease and concurrent COVID-19. He openly discussed his concerns and anxiety of cancer recurrence, his future, and his problems of going back home, alone and isolated. He repeatedly mentioned the uncertainty of his cancer prognosis and the fear of dealing with the surgical outcomes, mainly the impaired ability to eat and speak. Furthermore, his consideration of the future was worsened by the consciousness of being infected by SARS-CoV-2, deteriorating his mood with consequent psychological consequences, thus prompting a mandatory psycho-oncological support. During the first psycho-oncological session, we assessed his need of support, since recent evidences show that well-being requires a comprehensive health-related quality of life assessment (28).

As the patient expressed anxiety and fears on the consequences of COVID-19 test positivity, we decided to offer two psychological sessions per week. The aim was to provide support for the hospitalization distress, exacerbated by loneliness and the viral infection.

He reported his anxiety due to confinement, loss of usual routine, and reduced social and physical contact with others. This condition caused boredom, frustration, and sense of isolation from the rest of the world. His feelings were exacerbated by his inability to speak well, which also limited the use of devices for remote communication, such as video calls on phone or tablet, used for worldwide connection with friends and family in the pandemic time (29).

The patient's awareness of his vulnerability was the starting point for the psychological support. The psychologist together with the patient planned a daily schedule of activities, in order to decrease boredom, loss of routine, and helplessness. With time, the patient showed increased locus of control and self-regulation by organizing activities and managing well his time (self-care, video call with football team, friends). Unfortunately, 3 weeks after surgery he tested positive for COVID-19 again.

The patient was discharged on the 23rd postoperative day and was asked to maintain self-isolation as required by national health dispositions.

Upon discharge after such complex procedures such as head and neck surgery, patients are usually happy and eager to return home. On the contrary, in this case, the idea of discharge increased the patient's concerns. He emphasized the fear of infecting his family members and to be alone and was worried that this infection could affect his postoperative course (29).

Because of his condition, the psychological support proceeded even after discharge, with interviews scheduled regularly twice a week. The counseling sessions were arranged by video calls. As described, the home quarantine strongly affected the patient's psychological distress, more than the cancer itself (30). This profound anxiety and depression disturbed the patient during his convalescence, until the long-expected negative swab, when the patient was finally allowed to embrace his family.

These events led us to reflect on the importance of a psychological support for all surgical patients during the pandemic era.

At the referral Hospital, as reported by international guidelines, psychological support is routinely provided to patients affected by head and neck cancer, especially to patients who have undergone complex and demolitive surgical treatment (31), such as the one herein presented.

Previously, in the non-COVID era, during hospital stay, psychological support generally consisted in two or three meetings, when requested by the patient. These meetings are focused on the impact of demolitive surgery on the patient's future and quality of life. In case of a COVID-positive patient, all issues related to the impact of the oncological disease on life are amplified because of COVID-19 diagnosis. Indeed, these patients are faced with two diagnosis: cancer and the SARS-CoV-2 infection. Moreover, we are planning to compare the psychological status of patients with oral tongue cancer who developed COVID-19 to others without COVID-19 in a future study.

The major limitation of this prospective work is that it is a simple case report, with results that cannot be neither generalized nor easily applicable in a wider patient cohort.

However, we underline that case reports on oncological matters also have a role in medical education, inspiring profound scientific research questions for clinicians and scientists. This is especially relevant in this scenario, where tongue tumors are a rare oncological pathology and, as such, even a single case can be very instructive for clinicians. Furthermore, this case report highlights the importance of psychological support and should be tailored to the new needs of each patient in this current pandemic era.

Most likely, the psychological support should be anticipated in order to prepare oncological patients in managing their life postsurgery and treatment, by focusing not only on their cancer disease but also on a possible SARS-CoV-2-related condition. From now on, the preoperative patient's interview should be implemented by explaining how the new health conditions might affect their treatments and recovery on the top of the oncological disease.

The use of telemedicine may be a valid support to keep in contact with our fragile patients both during COVID-induced isolation and after discharge. A remote-controlled technologic device is an essential tool today to be able to focus on patients' mental health and well-being in this COVID-19 time. In fact, this first case occurring at the onset of the Italian pandemic era prompted in our hospital the structuring of psychological tele-visits to provide methodical health support for all COVID-positive cancer patients.

In conclusion, the psycho-oncological support could play a crucial role in many aspects of head and neck cancer patients' care. It may need to be adapted and enhanced in this pandemic period. These reflections lead to interesting suggestions toward the elaboration of future preventing strategies in times of stress and crisis for the healthcare system.

## Data Availability Statement

The raw data supporting the conclusions of this article will be made available by the authors, without undue reservation.

## Ethics Statement

Ethical review and approval was not required for the study on human participants in accordance with the local legislation and institutional requirements. The patients/participants provided their written informed consent to participate in this study. Written informed consent was obtained from the individual(s) for the publication of any potentially identifiable images or data included in this article.

## Author Contributions

MT conceptualized and designed the study. RD and PG drafted the initial manuscript and carried out and reviewed the literature review. FC and GPi revised the manuscript. SC, MA, GPr, and FM critically reviewed the manuscript for important intellectual content. All authors approved the final manuscript as submitted and agreed to be accountable for all aspects of this work.

## Conflict of Interest

The authors declare that the research was conducted in the absence of any commercial or financial relationships that could be construed as a potential conflict of interest.

## References

[1] World Health Organization. Coronavirus Disease 2019 (COVID 2019) Situation Report-90. Available online at: https://covid19.who.int/ (accessed April 19, 2021).

[2] Ansarin M. Surgical management of head and neck tumours during the SARS-CoV (Covid-19) pandemic. Acta Otorhinolaryngol Ital. (2020) 40:87–9. 10.14639/0392-100X-N0783 32271745 PMC7256907

[3] Al-Quteimat OM Amer AM. The impact of the COVID-19 pandemic on cancer patients. Am J Clin Oncol. (2020) 43:452–5. 10.1097/COC.0000000000000712 32304435 PMC7188063

[4] Vivarelli S Falzone L Grillo CM Scandurra G Torino F Libra M. Cancer management during COVID-19 pandemic: is immune checkpoint inhibitors-based immunotherapy harmful or beneficial?" *Cancers*. (2020) 8:2237. 10.3390/cancers12082237 PMC7465907 32785162

[5] Quaquarini E Saltalamacchia G Presti D Caldana G Tibollo V Malovini A. Impact of COVID-19 outbreak on cancer patient care and treatment: data from an outpatient oncology clinic in Lombardy (Italy). Cancers. (2020) 12:2941. 10.3390/cancers12102941 33053782 PMC7601200

[6] Brivio E Guiddi P Scotto L Giudice AV Pettini G Busacchio D. Patients living with breast cancer during the coronavirus pandemic: the role of family resilience, coping flexibility, and locus of control on affective responses. Front Psychol. (2021) 11:567230. 10.3389/fpsyg.2020.567230 33519580 PMC7840521

[7] Brivio E Oliveri S Pravettoni G. Empowering communication in emergency contexts: reflections from the italian coronavirus outbreak. Mayo Clin Proc. (2020) 95:849–51. 10.1016/j.mayocp.2020.03.021 32370848 PMC7252024

[8] Monroy-Iglesias MJ Tagliabue M Dickinson H Roberts G De Berardinis R Russell B. Continuity of cancer care: the surgical experience of two large cancer hubs in London and Milan. Cancers. (2021) 13:1597. 10.3390/cancers13071597 33808375 PMC8036608

[9] Arrangoiz R Cordera F Caba D Moreno E de León EL Muñoz M. Oral tongue cancer: literature review and current management. Cancer Rep Rev. (2018) 2:1–9. 10.15761/CRR.1000153

[10] Adjei Boakye E Johnston KJ Moulin TA Buchanan PM Hinyard L Tobo BB. Factors associated with head and neck cancer hospitalization cost and length of stay-a national study. Am J Clin Oncol. (2019) 42:172–8. 10.1097/COC.0000000000000487 30300170

[11] Koster ME Bergsma J. Problems and coping behavior of facial cancer patients. Soc Sci Med. (1990) 30:569–78. 10.1016/0277-9536(90)90155-L 2408153

[12] Björklund M Sarvimäki A Berg A. Living with head and neck cancer: a profile of captivity. J Nurs Healthc Chronic Illn. (2010) 2:22–31. 10.1111/j.1752-9824.2010.01042.x

[13] Ward EC van As-Brooks CJ. Head and Neck Cancer: Treatment, Rehabilitation and Outcomes. San Diego: Pleural Publishing, Inc. (2007). p. 1–640.

[14] Howren MB Christensen AJ Karnell LH Funk GF. Psychological factors associated with head and neck cancer treatment and survivorship: evidence and opportunities for behavioral medicine. J Consult Clin Psychol. (2013) 81:299–317. 10.1037/a0029940 22963591 PMC3587038

[15] Amin MB Edge SB Greene FL. AJCC Cancer Staging Manual. 8th ed. New York, NY: Springer (2017). p. 55–184.

[16] NCCN. National Comprehensive Cancer Network Guidelines. (2020). Available online at: http://www.nccn.org/professionals/physician_gls/pdf/head-and-neck.pdf (accessed April 3, 2021).

[17] Lee-Jones C Humphris G Dixon R Hatcher M.B. Fear of cancer recurrence–a literature review and proposed cognitive formulation to explain exacerbation of recurrence fears. Psychooncology. (1997) 6:95–105. 10.1002/(SICI)1099-1611(199706)6:2 9205967

[18] Simard S Savard J Ivers H. Fear of cancer recurrence: specific profiles and nature of intrusive thoughts. J Cancer Surviv. (2010) 4:361–71. 10.1007/s11764-010-0136-8 20617394

[19] Folkman S Lazarus RS. Stress, Appraisal, and Coping. New York, NY: Springer Publishing Company (1984). p. 150–153.

[20] Bachmann AS Zaunbauer AC Tolke AM Siniatchkin M Kluck C Wiltfang J. Well-being and quality of life among oral cancer patients–Psychological vulnerability and coping responses upon entering initial treatment. J Craniomaxillofac Surg. (2018) 46:1637–44. 10.1016/j.jcms.2018.05.042 29960813

[21] Bloom JR. Social support, accommodation to stress and adjustment to breast cancer. Soc Sci Med. (1982) 16:1329–38. 10.1016/0277-9536(82)90028-4 7123275

[22] Marroquín B Czamanski-Cohen J Weihs KL Stanton AL. Implicit loneliness, emotion regulation, and depressive symptoms in breast cancer survivors. J Behav Med. (2016) 39:832–44. 10.1007/s10865-016-9751-9 27287618 PMC5014704

[23] Blake-Mortimer J Gore-Felton C Kimerling R Turner-Cobb JM Spiegel D. Improving the quality and quantity of life among patients with cancer: a review of the effectiveness of group psychotherapy. Eur J Cancer. (1999) 35:1581–86. 10.1016/S0959-8049(99)00194-X 10673965

[24] Renzi C Fioretti C Oliveri S Mazzocco K Zerini D Alessandro O. A qualitative investigation on patient empowerment in prostate cancer. Front Psychol. (2017) 8:1215. 10.3389/fpsyg.2017.01215 28798701 PMC5526923

[25] Oliveri S Arnaboldi P Pizzoli S Faccio F Giudice AV Sangalli C. PTSD symptom clusters associated with short- and long-term adjustment in early diagnosed breast cancer patients. Ecancermedicalscience. (2019) 13:917. 10.3332/ecancer.2019.917 31123500 PMC6467457

[26] Masiero M Busacchio D Guiddi P Arnaboldi P Musi G De Cobelli O. Quality of life and psycho-emotional well-being in bladder cancer patients and their caregivers: a comparative analysis between urostomy versus ileal orthotopic neobladder. Ecancermedicalscience. (2021) 15:1163. 10.3332/ecancer.2021.1163 33680077 PMC7929779

[27] Seligman ME Maier SF. Failure to escape traumatic shock. J Exp Psychol. (1967) 74:1–9. 10.1037/h0024514 6032570

[28] Happ MB Roesch T Kagan SH. Communication needs, methods, and perceived voice quality following head and neck surgery: a literature review. Cancer Nurs. (2004) 27:1–9. 10.1097/00002820-200401000-00001 15108946

[29] Brooks S Webster R Smith L. The psychological impact of quarantine and how to reduce it: rapid review of the evidence. Lancet. (2020) 395:912–20. 10.1016/S0140-6736(20)30460-8 32112714 PMC7158942

[30] National Comprehensive Cancer Network. Guidelines for patients. Distress During Cancer Care. (2020). Available online at: https://www.nccn.org/patients/guidelines/cancers.aspx (accessed April 3, 2021).

[31] Arnaboldi P Oliveri S Vergani L Marton G Guiddi P Busacchio D. The clinical-care focused psychological interview (CLiC): a structured tool for the assessment of cancer patients' needs. Ecancermedicalscience. (2020) 14:1000. 10.3332/ecancer.2020.1000 32153655 PMC7032941

